# Supplementary figures and images for: Collective dynamics of strain-coupled nanomechanical pillar resonators
Source: Nat Commun. 2019 Nov 20;10:5246. doi: 10.1038/s41467-019-13309-9 (PMC6868224; doi:10.1038/s41467-019-13309-9)

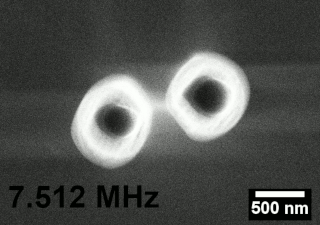

Supplement: Supplementary file 1 — Supplementary Movie 1 [file 41467_2019_13309_MOESM1_ESM.gif]

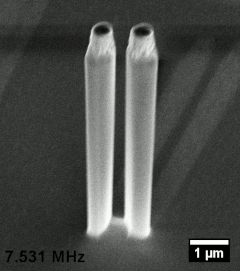

Supplement: Supplementary file 2 — Supplementary Movie 2 [file 41467_2019_13309_MOESM2_ESM.gif]
