# Supplementary material for: Collective dynamics of strain-coupled nanomechanical pillar resonators
Source: Nat Commun. 2019 Nov 20;10:5246. doi: 10.1038/s41467-019-13309-9 (PMC6868224; doi:10.1038/s41467-019-13309-9)
Supplement: Supplementary file 3 — Supplementary Information [file 41467_2019_13309_MOESM3_ESM.pdf]

Supplementary Information: Collective dynamics of strain-coupled nanomechanical pillar resonators

J. Doster[1], S. Hoenl[1,*], H. Lorenz[2], P. Paulitschke[2] & E. M. Weig[1]

[1] *Department of Physics, University of Konstanz, Universitätsstrae 10, 78457 Konstanz, Germany*

[2] *Fakultät für Physik and Center for NanoScience (CeNS), Ludwig-Maximilians-Universität, Geschwister-Scholl-Platz 1, 80539 München , Germany*

[*] Present address: IBM Research Zurich, Säumerstrasse 4, CH-8803 Rüschlikon, Switzerland

Supplementary Note 1: Overview

In Supplementary Note 2, we discuss supplementary measurements on the nanopillar pair shown in Fig. 2 of the main text, including scanning electron microscope measurements as well as a characterization in the laser setup. A thorough description of the thermal tuning mechanism is found in Supplementary Note 3. In Supplementary Note 4, the simulation model and data processing to numerically evaluate the pillar coupling is described in more detail. In Supplementary Note 5, we show numerical simulations and experimental data for different orientations of the pillar pair on the substrate.

Supplementary Note 2: Supplementary measurements

In this section, we show the full experimental characterization of the nanopillar pair investigated in Fig. 2 of the main text ($r \approx 310\,\mathrm{nm}$, $H \approx 7\,\mu\mathrm{m}$, $d \approx 1.3\,\mu\mathrm{m}$). Supplementary Figure 1a shows a scanning electron micrograph of the nanopillar pair at rest. Comparing this to Supplementary Fig. 1c-f, one can clearly identify four different modes belonging to the four fundamental flexural modes of the two nanopillars, RH, LH, LV, and RV. While for two identical, perfectly circular pillars all eigenfrequencies are degenerate, fabricational imperfections give rise to the observed frequency differences. A sweep of the drive frequency f_{drive} confirms that there is no spectral overlap between the modes, and that their frequency separation is roughly $10 \times \Delta f$. The slight vibration of the right pillar in Supplementary Fig. 1d can therefore be interpreted as a beginning hybridization between the two pillars as described in Fig. 2 of the main text. Even though the

two pillars are not on resonance (since their eigenfrequencies do not coincide as discussed above), driving the left pillar gives rise to an excitation of the right pillar as a result of the inter-pillar coupling.

A more complete understanding is obtained by frequency tuning the pillars on resonance. This is accomplished by thermal tuning of the right (higher frequency) pillar (see 3) in the optical detection setup. The resulting avoided crossing is shown in Supplementary Fig. 1b, along with a fit of the model [1] (see Supplementary Note 4). The resulting level splitting yields a coupling strength of $g/2\pi = 33.3(30)\,\mathrm{kHz}$, which clearly exceeds the linewidth $\Delta f \approx 3\,\mathrm{kHz}$, confirming the strong coupling between the two nanopillars.

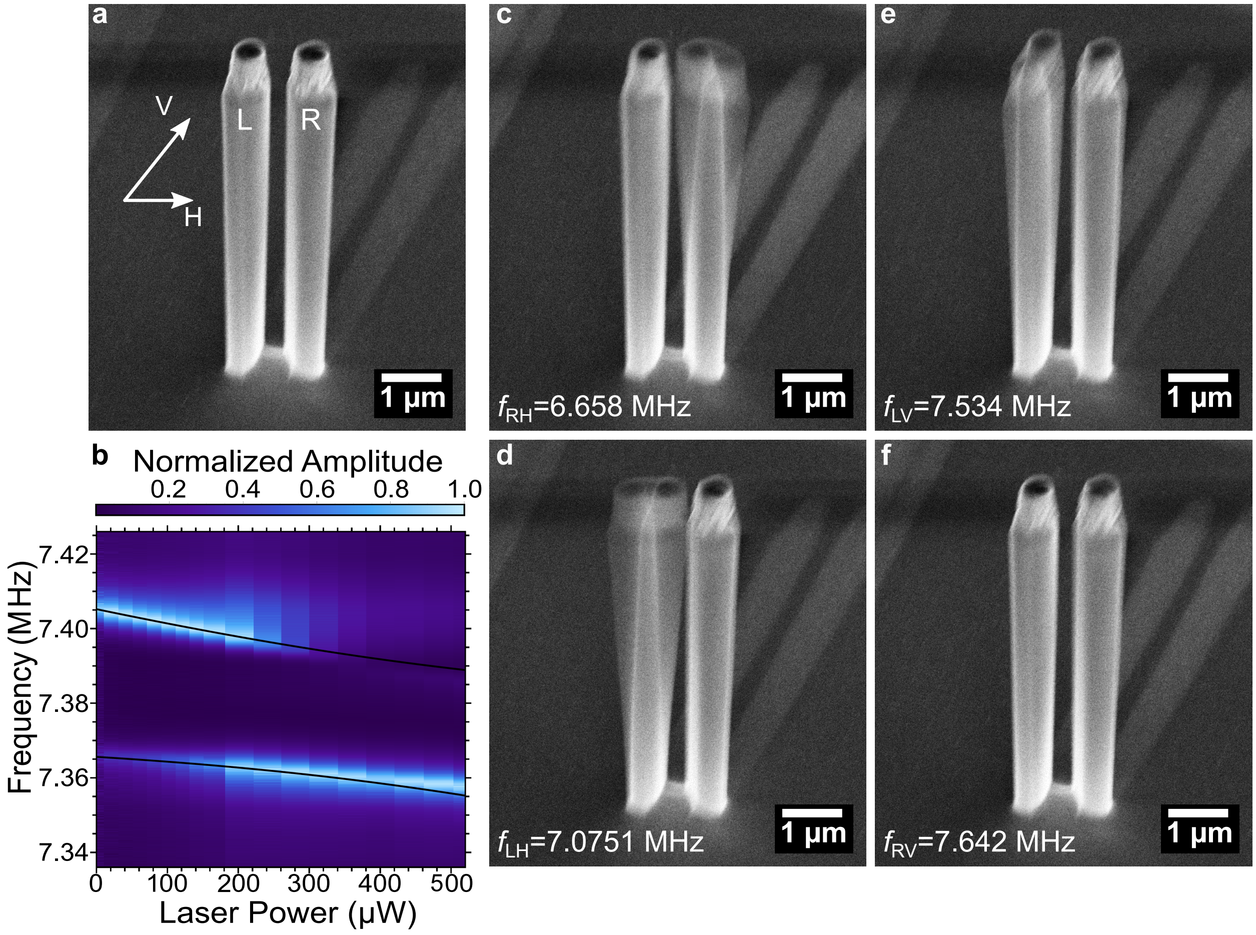

Supplementary Figure 1: ***Characterization of the nanopillar pair in Fig. 2** **(**$r \approx 310\,\mathrm{nm}$**,** $H \approx 7\,\mu\mathrm{m}$**,** $d \approx 1.3\,\mu\mathrm{m}$**).** **a**, Scanning electron micrograph of the two undriven resonators. **b**, Optically detected avoided crossing of the same nanopillar pair under thermal tuning of the right pillar. Fit to the data (black) yields a level splitting of* $g/2\pi = 33.3(30)\,\mathrm{kHz}$*.* ***c**, **d**, **e**, **f**, scanning electron micrographs of the four distinct eigenmodes RH, LH, LV, and RV of the nanopillar pair at the indicated driving frequency.*

Supplementary Note 3: Tuning mechanism

In the optical detection setup, the nanopillar vibration is probed by the reflection of a laser with $\lambda = 635\,\mathrm{nm}$ focused on the nanopillar from above. Since gallium arsenide absorbs roughly $1-R = 65\,\%$ [2] of the red laser light, this induces a certain amount of heating of the nanopillar. Finite element simulation of a single nanopillar $r = 291\,\mathrm{nm}$, $H = 6.1\,\mu\mathrm{m}$ and $\varphi = 1.38°$ irradiated by red light with $700\,\mu\mathrm{W}$ laser power yields the temperature distribution along this nanopillar (see Supplementary Fig. 2a). Evidently, the nanopillar temperature can be increased by a few hundred degrees, depending on the nanopillar geometry and the laser power. For laser powers exceeding $1\,\mathrm{mW}$ irreversible damage and melting of the nanopillars have been experimentally observed.

Since the Young's modulus E of most materials is temperature dependent, laser-induced heating can be employed to reduce $E(T)$ and in turn the eigenfrequency. For the case of the inverted conical GaAs nanopillars investigated here, the flexural fundamental eigenfrequency ω_0 of these resonators is obtained by $\omega_0 = \sqrt{\frac{E}{\rho}}G(r, H, \varphi)$, with mass density ρ and a geometry factor $G(r, H, \varphi)$ derived in ref. [3]. Hence, irradiating the nanopillar with increasing laser power allows to continuously lower the resonance frequency of its flexural eigenmodes, similar to the thermal tuning of the optical transitions of quantum dots and cavities. [4–6] The thermal tuning of a single nanopillar as well as a theoretical prediction based on the simulated heating as in Supplementary Fig. 2a is shown in Supplementary Fig. 2b. For up to $500\,\mu\mathrm{W}$, excellent agreement is observed. The slight deviation of the experimentally observed frequency tuning from the theoretical prediction at $600\,\mu\mathrm{W}$ is attributed to a beginning degradation of the nanopillar.

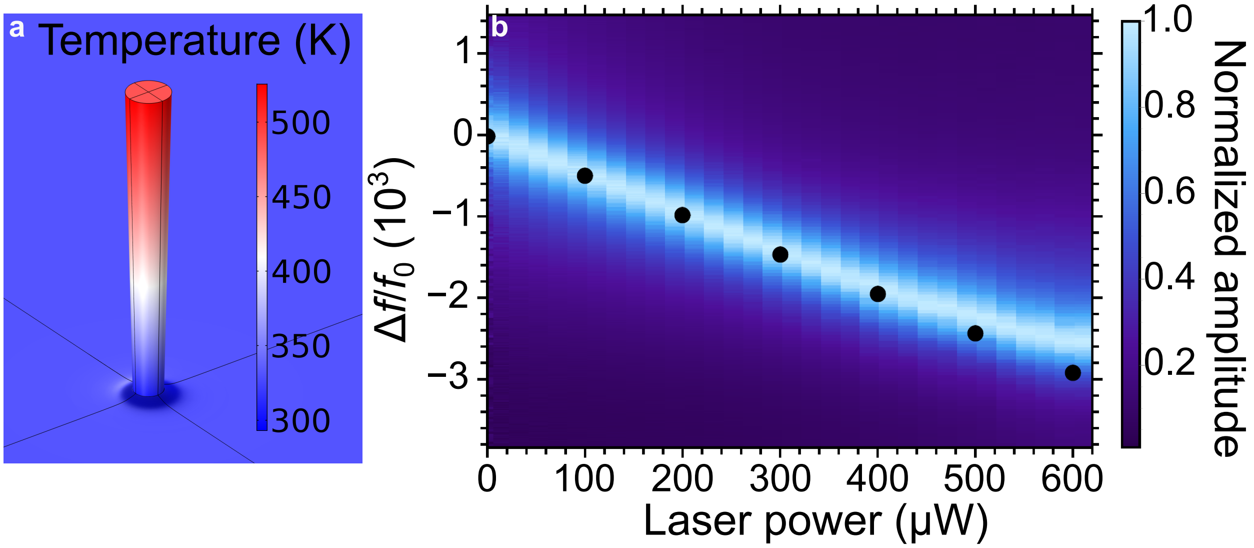

Supplementary Figure 2: ***Thermal tuning. a**, Finite element simulation of the temperature distribution inside the nanopillar subject to laser irradiation at* $\lambda = 635\,\mathrm{nm}$ *with* $700\,\mu\mathrm{W}$ *from the top.* ***b**, Measured frequency tuning* $\Delta f/f_0$ *of a single nanopillar's resonance frequency* f_0 *as a function of the laser power of the read-out laser. The corresponding simulation data are shown as black dots.*

Thermal tuning is applied to tune the two corresponding eigenmodes of a pillar pair (e.g. RV and LV) into resonance. To this end, the laser is focused on the nanopillar with the higher resonance frequency. Thus, its eigenfrequency is tuned towards the lower-lying eigenfrequency of the second nanopillar, which enables measuring the avoided level crossing (in case of strong coupling). For all figures displaying thermal tuning (Fig. 3, Supplementary Fig. 1 and Supplementary Fig. 2), the x-axis represents the laser power measured outside the vacuum chamber after passing through a beam splitter. Note that a constant offset arising from an illuminating LED used for the positioning

of the nanopillar pair has been subtracted from the measured power. Since the LED is not focused on the tuned pillar, the resulting frequency offset is small and applies to all pillars in the field of view. As a result of the laser being focused on one nanopillar, the displacement signal of that pillar is much stronger than that of the second pillar which arises solely from its interaction with the stray field of the laser. This allows to distinguish the response from the two nanopillars, and also entails a further indication for hybridization, when the response of both modes becomes approximately equal.

Supplementary Note 4: Simulation data of frequency tuning and coupling strength

Our measurements are guided and supported by results from finite element simulations. Figure 5 of the main text as well as Supplementary Fig. 4a show the simulation model of a nanopillar pair. In particular, the nanopillar foot as well as the ridge formed between the two pillars has to be carefully modeled to match the geometry of the experimentally explored nanopillars (see Fig. 5b) for an accurate prediction of the elastic properties of the nanopillar pair.

To simulate the coupling strength, the thermal tuning mechanism is emulated by a parametric sweep of the Young's modulus $E + \Delta E$ of one nanopillar corresponding to the laser-irradiated nanopillar in the measurement. As in Supplementary Note 3 the resonance frequency of this nanopillar tunes towards lower frequencies with lower ΔE. We assume an isotropic substrate with the Young's modulus of the [100] crystal direction, $E_{[100]} = 85.9\,\mathrm{GPa}$.

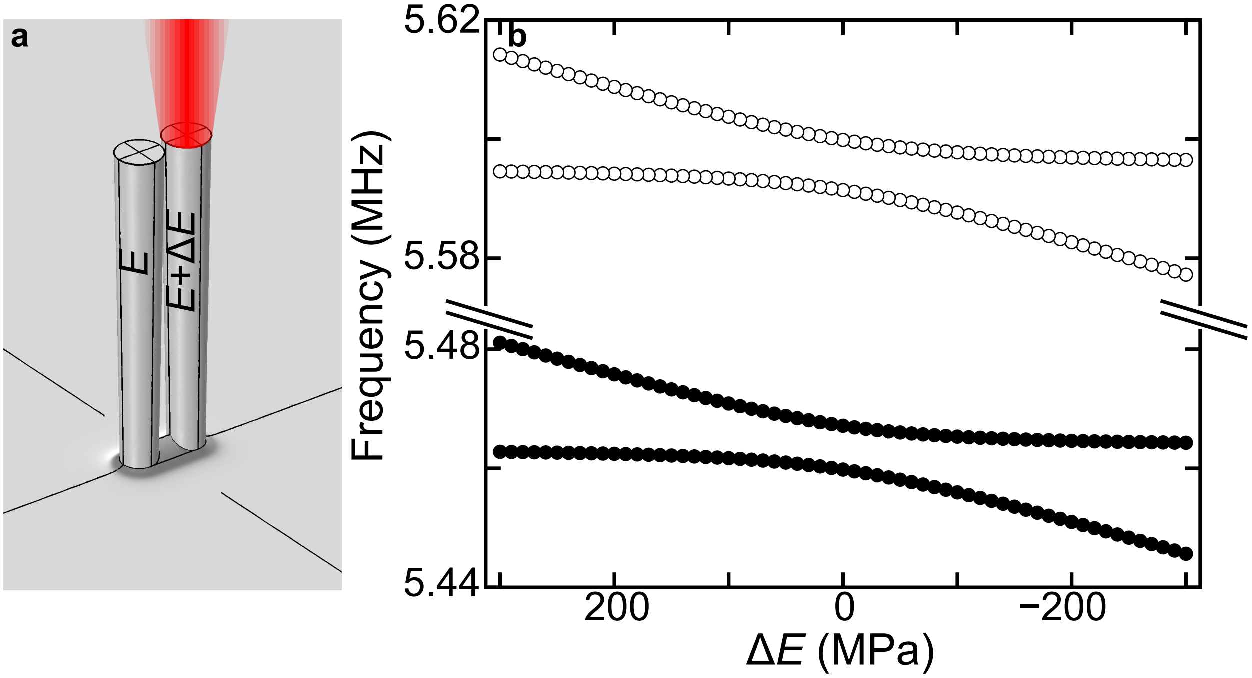

Supplementary Figure 3: ***Simulated avoided crossing.*** ***a****, Finite element simulation model, indicating the laser tuning of the right nanopillar by a laser beam with* $\lambda = 635\,\mathrm{nm}$. *In the simulation the tuning is realized by a parametric sweep of the Young's modulus of the right nanopillar* $E + \Delta E$. *For illustration a laser beam (red) has been added.* ***b****, Simulation results, showing four modes separated in doublets as a result of the anisotropy of the nanopillar foot (see Fig. 1c, main text).* ● *(*○*) represents vertical (horizontal) mode polarization with respect to the nanopillar connecting line, respectively. Each of the doublets shows an avoided crossing.*

The simulation data in Supplementary Fig. 4b reveal four eigenmodes, separating into two sets of two resonance frequencies. The frequency splitting is a consequence of the anisotropy of the nanopillar clamping point, leading to a different stiffness of the eigenmodes vibrating along and perpendicular to the connecting line of the nanopillars. Assuming that the nanopillar pair is aligned

along the [110] crystal direction, the slightly stiffer modes along the connecting line indicated by white circles are assigned to the horizontal (H), while the slightly softer modes perpendicular to the connecting line denoted by black circles are identified with the vertical (V) vibration polarization. Each of the two sets exhibits an avoided crossing, which indicates strong coupling between the LH and the RH, as well as the LV and the RV mode, respectively. The frequency splitting between the H and V modes is also observed for the experimentally investigated nanopillar pairs. It does not arise in simulations of a nanopillar pair on a flat substrate. We therefore conclude that the large observed frequency splittings are inherent to nanopillar pairs, and can be ascribed to the broken symmetry of the pillar foot for that particular geometric configuation. Note that this implies that the observed frequency differences exceed the net frequency disorder resulting from fabrication imperfections. An assessment of the frequency disorder is therefore not possible for nanopillar pairs.

To analyze the simulated and measured avoided crossings we use the model of two linearly coupled harmonic oscillators A and B. It assumes the two coupled differential equations [1]

$$m_A \ddot{x}_A + k_A x_A + \kappa(x_A - x_B) = 0 \tag{1}$$

$$m_B \ddot{x}_B + k_B x_B - \kappa(x_A - x_B) = 0 \tag{2}$$

with respective effective masses m_i, spring constants k_i and coupling spring constant κ. Solving this model, gives rise to the level splitting

$$g = \frac{\sqrt{\kappa/m_A}\sqrt{\kappa/m_B}}{\sqrt{\omega_A \omega_B}} \tag{3}$$

which depends on the coupling spring constant κ and the eigenfrequencies of the respective res-

onators ω_i, and defines the coupling strength in units of frequency.

The simulated coupling rates are discussed in Fig. 5 of the main text. Very similar coupling rates are found for the horizontally and vertically polarized modes. Interestingly, this is not the case for the nanopillar pair on a flat substrate, for which the horizontal modes exhibit a coupling rate which exceeds that of the vertical modes by almost a factor of 2. This suggests that the broken symmetry of the pillar foot homogenizes the coupling rates for the two mode polarizations.

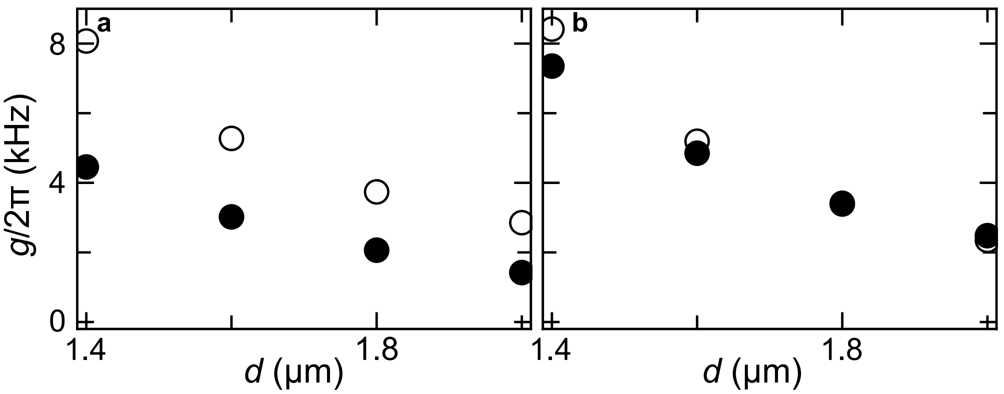

Supplementary Figure 4: ***Coupling strength and clamping geometry.*** *Simulated coupling rate of a typical nanopillar pair (*$r = 400\,nm$*,* $H = 7\,\mu m$*, taper angle* $1°$*) as a function of center-to-center distance.* ***a****, Nanopillar pair on a flat substrate.* ***b****, Nanopillar pair with realistic clamping conditions.* ● *(*○*) represents vertical (horizontal) mode polarization with respect to the nanopillar connecting line, respectively.*

Supplementary Note 5: Angular dependence of the coupling strength

Supplementary Figure 5a shows the theoretical angular dependence of the Young's Modulus on a (100) GaAs substrate. [7,8] Correspondingly, a variation of the orientation of the pillar pair is expected to lead to a change in the strain-mediated inter-pillar coupling. Following this consideration, Supplementary Fig. 5b shows the simulated coupling rates when the Young's Modulus of an (isotropic) substrate is changed according to the theoretical value along the connecting line of the pillars. The simulation data shows a clear angular dependence indicating that a smaller Young's modulus in the substrate increases the coupling strength and thus, promotes the energy transfer between the pillars. As already pointed out in the main text, the data in Fig. 4a has been obtained for different orientation angles of the pillar pair. The angles are indicated as different symbols in Supplementary Fig. 5c, which otherwise repeats the data from Fig. 4a. A clear correlation between the coupling strength and the angle is not apparent, and the hypothesis extracted from numerical simulation can not be validated, presumably as a result of the large scatter in the experimentally obtained coupling rates.

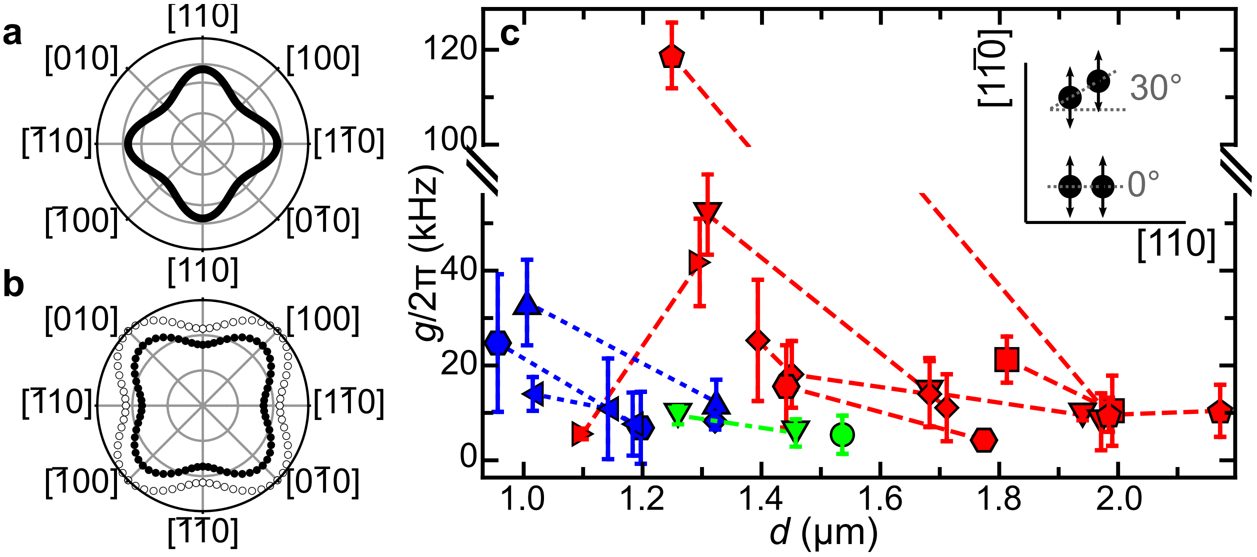

Supplementary Figure 5: ***Geometry dependence of coupling strength. a**, Theoretical angular dependence of the Young's Modulus on a (100) GaAs wafer calculated from* [7,8]. ***b**, Simulated level splittings of vibrational modes orthogonal and along the connecting line (●, ○, respectively), assuming an isotropic substrate which the Young's Modulus corresponding to the angular orientation of the pillar pair. **c**, Experimentally determined coupling rate* $g/2\pi$ *of the vertical modes of the two nanopillars over their center-to-center distance* d *for several samples and pillar pair geometries with* $r \approx 430\,\mathrm{nm}$ *&* $H \approx 7\,\mu\mathrm{m}$ *(red, dashed),* $r \approx 335\,\mathrm{nm}$ *&* $H \approx 7\,\mu\mathrm{m}$ *(blue, dotted),* $r \approx 330\,\mathrm{nm}$ *&* $H \approx 8.2\,\mu\mathrm{m}$ *(green, dotted-dashed). In addition, filled symbols indicate the orientation of the pillar pair with respect to the [110] crystal direction,* $0°$ *(●),* $10°$ *(■),* $15°$ *(▲),* $20°$ *(▼),* $25°$ *(⬟),* $30°$ *(◆),* $35°$ *(⬣),* $40°$ *(▶) and* $45°$ *(◀).*

Supplementary References

1. Novotny, L. Strong coupling, energy splitting, and level crossings: A classical perspective. *American Journal of Physics* **78**, 1199–1202 (2010). URL https://doi.org/10.1119/1.3471177.

2. Aspnes, D. E., Kelso, S. M., Logan, R. A. & Bhat, R. Optical properties of $As_xGa_{1-x}As$. *Journal of Applied Physics* **60**, 754–767 (1986). URL https://aip.scitation.org/doi/10.1063/1.337426.

3. Paulitschke, P., Seltner, N., Lebedev, A., Lorenz, H. & Weig, E. M. Size-independent Youngs modulus of inverted conical GaAs nanowire resonators. *Applied Physics Letters* **103**, 261901 (2013). URL https://doi.org/10.1063/1.4851897.

4. Kiraz, A. *et al.* Cavity-quantum electrodynamics using a single InAs quantum dot in a microdisk structure. *Applied Physics Letters* **78**, 3932–3934 (2001). URL https://doi.org/10.1063/1.1379987.

5. Reithmaier, J. P. *et al.* Strong coupling in a single quantum dot-semiconductor microcavity system. *Nature* **432**, 197–200 (2004). URL https://doi.org/10.1038/nature02969.

6. Yoshie, T. *et al.* Vacuum Rabi splitting with a single quantum dot in a photonic crystal nanocavity. *Nature* **432**, 200–203 (2004). URL https://doi.org/10.1038/nature03119.

7. Levinshtein, M., Rumyantsev, S. & Shur, M. *Handbook Series on Semiconductor Parameters - Volume 2: Ternary And Quaternary III-V Compounds*, vol. 2 (WORLD SCIENTIFIC, 1996). URL `http://www.ioffe.ru/SVA/NSM`.

8. Hopcroft, M. A., Nix, W. D. & Kenny, T. W. What is the Young's Modulus of Silicon? *Journal of Microelectromechanical Systems* **19**, 229–238 (2010). URL `https://doi.org/10.1109/JMEMS.2009.2039697`.
