# Supplementary material for: Collective dynamics of strain-coupled nanomechanical pillar resonators
Source: Nat Commun. 2019 Nov 20;10:5246. doi: 10.1038/s41467-019-13309-9 (PMC6868224; doi:10.1038/s41467-019-13309-9)
Supplement: Supplementary file 5 — Description of Additional Supplementary Files [file 41467_2019_13309_MOESM5_ESM.pdf]

Title: Supplementary Movie 1:
Description: Mode hybridization. Scanning electron micrograph of a pair of nanopillars (H≈7 µm, r≈310 nm, d≈1.3 µm) imaged from the top. The pair is driven near the vertical resonance frequency of the left pillar (fdrive≈fLV). Inset shows the varying drive frequency.

Title: Supplementary Movie 2:
Description: Mode hybridization. Scanning electron micrograph of a pair of nanopillars (H≈7 µm, r≈310 nm, d≈1.3 µm) imaged in a 60° tilted view. The pair is driven near the vertical resonance frequency of the left pillar (fdrive≈fLV). Inset shows the varying drive frequency.
